# A pH-Sensitive, Biobased Calcium Carbonate Aragonite Nanocrystal as a Novel Anticancer Delivery System

**DOI:** 10.1155/2013/587451

**Published:** 2013-11-14

**Authors:** Abdullahi Shafiu Kamba, Maznah Ismail, Tengku Azmi Tengku Ibrahim, Zuki Abu Bakar Zakaria

**Affiliations:** ^1^Laboratory of Molecular Biomedicine, Institute of Bioscience, Universiti Putra Malaysia, 43400 Serdang, Malaysia; ^2^Faculty of Veterinary Medicine, Universiti Putra Malaysia, 43400 Serdang, Malaysia

## Abstract

The synthesised biobased calcium carbonate nanocrystals had demonstrated to be an effective carrier for delivery of anticancer drug doxorubicin (DOX). The use of these nanocrystals displayed high levels of selectivity and specificity in achieving effective cancer cell death without nonspecific toxicity. These results confirmed that DOX was intercalated into calcium carbonate nanocrystals at high loading and encapsulation efficiency (4.8 and 96%, resp.). The CaCO_3_/DOX nanocrystals are relatively stable at neutral pH (7.4), resulting in slow release, but the nanocrystals progressively dissociated in acidic pH (4.8) regimes, triggering faster release of DOX. The CaCO_3_/DOX nanocrystals exhibited high uptake by MDA MB231 breast cancer cells and a promising potential delivery of DOX to target cells. *In vitro* chemosensitivity using MTT, modified neutral red/trypan blue assay, and LDH on MDA MB231 breast cancer cells revealed that CaCO_3_/DOX nanocrystals are more sensitive and gave a greater reduction in cell growth than free DOX. Our findings suggest that CaCO_3_ nanocrystals hold tremendous promise in the areas of controlled drug delivery and targeted cancer therapy.

## 1. Introduction

Approximately 90% of cancer fatalities are as a result of metastatic cancer spread to vital organs, leading to complications such as hypercalcaemia, pain, cord compression, pathological fracture, and anaemia, rather than being caused by the cancer at the primary tumour site [1, 2]. Bone is the most frequent site for distant metastasis in women with breast cancer [3, 4], with a reported incidence of up to 75% and an average survival time of approximately 2 years after diagnosis [5]. Bone metastases are the leading cause of morbidity and mortality of patients with breast cancer [6, 7]. The metastasis of breast tumour into bone cancers follows Paget's seed and soil theory, with bone marrow acting as fertile soil and cancer as the seeds; the bone marrow is providing stimulatory factors for the development and progression of  bone metastases [6], including resorption of tumour cells and the proliferation of angiogenic factors which are important in crosstalk between bone cells (osteoclasts and osteoblasts) and endothelial bone marrow cells [4]. Metastases of cancer to the bone represent the final, most devastating stage of malignancy and are the leading cause of death. Breast cancer is incurable after it has metastasized to bone, while bone metastasis can increase the rate of progression, and generates novel metastases in soft tissues [1, 2]. Therefore, the fundamental strategies for managing bone cancer metastasis are to understand the molecular mechanisms that would provide more antagonistic approach to prevent the development of  bone metastases as well as to treat the established metastatic bone lesions.

The current cancer therapies include surgery, hormonal therapy, radiation, and chemotherapy, with each being employed depending on the nature of the cancer and its extent of progression. In particular, chemotherapy is the standard method of treatment for breast cancer [5]. Chemotherapeutic agents are classified based on their structure and mode of action into the following groups: anthracycline, alkaloids, topoisomerase inhibitors, alkylating agents, and antimetabolites [6]. These therapeutic agents are used to suppress cell division and inhibit cancer proliferation, but they often lack specificity and selectivity, as well as affecting both cancerous and normal cells; this nonspecificity of cancer chemotherapies may result in a range of cumulative and life-threatening side effects, such as cardiac toxicity, neuropathy, neutropenia, kidney failure, nausea, and hair loss [7, 8]. These dangerous side effects limit the dose that can be applied to tumour cells.

Doxorubicin (DOX) is one of the therapeutically effective anticancer drugs belonging to a family of anthracycline agents, approved for the treatment of tumours. DOX acts through the integration of its structure between the base pairs of DNA or through the inhibition of topoisomerase II by preventing DNA synthesis [9, 10]. However, the major drawback which limits the usage of Dox is its toxicity [11].

Currently, nanomedicine delivery systems show great promise in mitigating the shortcomings of conventional chemotherapy by increasing drug solubility, specific tumour targeting, enhanced accumulation in tumour tissue and tumour cells, reducing the drugs side effect to normal cells (reduce the potential of non-specific toxicity) and increasing maximum tolerated dosage (allowing the use of a lower dose to the target site). Nanodrugs can selectively accumulate in tumours through a passive targeting mechanism known as the enhanced permeability and retention (EPR) effect [11].

The purpose of the current study is to develop an efficient drug delivery system and investigate the molecular mechanism for the enhanced cytotoxicity induced by DOX-loaded nanocrystals. To the best of our knowledge, no research has been reported pertaining to the use of biobased calcium carbonate nanocrystals derived from cockle shells (*Anadara granosa*), in which the primary component is calcium carbonate (98-99%) [12]. We are specifically interested in the synthesis of a rod-shaped aragonite polymorph of calcium carbonate for drug delivery applications. One of the advantages of the aragonite polymorph is its greater comparative density than that of calcite, which makes it biocompatible with applications in the medical, pharmaceutical, cosmetic, and paint industries. 

In the current study, we used calcium carbonate as the main source of calcium and carbonate ions in contrast to the majority of previous researches, which focused on the use of chemical reactions to yield the calcium and carbonate precursors for the synthesis of calcium carbonate nanocrystals.

## 2. Materials and Methods

### 2.1. Synthesis of Calcium Carbonate Nanocrystals

Syntheses of calcium carbonate nanocrystals were carried out in oil-in-water (O/W) microemulsions using higher pressure homogeniser (HPH) as described in our published article [13]. In this technique, the particles sizes are reduced after leaving the homogenising gap by cavitations, particle collisions, and shear forces.

### 2.2. Characterisation of Calcium Carbonate Nanocrystal

 Particles size and morphology were characterised by transmission electron microscopy (TEM, Hitachi H-7100) and field emission scanning electron microscopy (FESEM, JOEL 7600F).

### 2.3. Elemental Analysis of CaCO_3_/Dox Nanocrystals and Pure Doxorubicin

Two (2 mg) of both CaCO_3_/Dox nanocrystals and pure doxorubicin were accurately weighed in a tin capsule and weighed twice once after filling the tin capsule and again after folding. The prepared samples are delivered by a rotating autosampler into a combustion furnace of CHNS-932 analyser from LECO Instruments (St Joseph, MI) with a pulse of pure oxygen. All combustible materials in the sample are combusted to gases; that is, all carbon-bearing materials are converted into CO_2_ and nitrogen-bearing materials to N_2_. The resulting combustion gases (product) are separated by GC column and measured by a thermal conductivity detector (TCD).

### 2.4. Preparation of Drug-Loaded Calcium Carbonate Nanocrystals

Doxorubicin hydrochloride (1 mg) was added to the calcium carbonate nanocrystal suspension (containing 50 mg of nanocrystals). The encapsulation of DOX in CaCO_3_ nanocrystals was achieved by continuous stirring of the suspension overnight, in a dark environment at room temperature. Calcium carbonate nanocrystals containing DOX were washed, centrifuged, and oven- dried (FD 115, Fisher Scientific, Germany) at 55°C.

### 2.5. Determination of Drug Loading and Encapsulation Efficiency

Drug loadings and encapsulation efficiencies of the CaCO_3_ nanocrystals were analysed by calculating the difference between the total of the drug feed (*W*_*t*_) and the free drug concentration in the supernatant liquid (*W*_*f*_) of the suspension per weight of CaCO_3_ nanocrystals. The amount of free DOX remaining in the supernatant solution was determined by measuring the absorbance at 485 nm with a UV-vis spectrophotometer (PerkinElmer Lambda 35 Boston, MA, USA). Data are reported as the average values of 3 independent measurements. Drug loading and encapsulation efficiency are calculated according to the “Pharmacopeia of People's Republic of China” (2000), as stated in the following:

(1) $$\begin{array}{c} \text{Loading}\;\text{content}=\frac{W_{t}-W_{f}}{W_{nP}}\times 100, \end{array}$$

where *W*_*t*_ is the total weight of drug fed, *W*_*f*_ is the weight of nonencapsulated free drug, and *W*_*nP*_ is the weight of the nanoparticles, and

(2) $$\begin{array}{c} \text{Encapsulation}\;\text{efficiency}=\frac{W_{t}-W_{f}}{W_{t}}\times 100, \end{array}$$

where *W*_*t*_ is the total weight of drug fed and *W*_*f*_ is the weight of non-encapsulated free drug.

### 2.6. *In Vitro* Drug Release

Drug-loaded calcium carbonate nanocrystal suspensions (containing approximately 20 mg of nanoparticles) in 50 mL of PBS buffer (pH 4.8 and 7.4) were prepared. Drug release concentrations were determined at specific intervals by measuring the absorbance at 480 nm using (UV-vis spectrophotometer PerkinElmer Lambda 35 Boston, MA, USA) connected to automated computer loaded with spectra manager software UV Probe. Data are reported as the mean ± standard deviation (SD) based on the three independent measurements.

### 2.7. *In Vitro* Evaluation of Cytotoxicity

Breast cancer cells, MDA MB231, were purchased from the American Type Culture Collection (ATCC) (Manassas, VA, USA) and cultured in a DMEM medium supplemented with 10% fetal bovine serum, L-glutamine (15 mmol/L), penicillin (100 U/mL), and streptomycin (100 *μ*g/mL). The culture was incubated in a 5% CO_2_ atmosphere at 37°C. Cells at 80%–90% confluence were used for seeding and treatment.

### 2.8. Cells Seeding and Treatment

The cells were seeded into a 96-well plate at a density of 0.5 × 10^4^ per well and incubated for 24 h. Cells were then cocultured with different concentrations of DOX and the CaCO_3_/DOX nanocrystals suspension (0 to 2 *μ*g/mL) for the periods of 24, 48, and 72 h. After the exposure was completed, the media was aspirated and washed with PBS before being replaced with additional media without serum prior to MTT treatment. Aliquots of 20 *μ*L of MTT reagent (Sigma Aldrich) in PBS were added into each well, and the plate was incubated at 37°C for 4 h (Thermo Fisher Scientific LPG). After this time, the culture medium was removed, and 200 *μ*L of Dimethyl sulfoxide (DMSO) was added into the wells. The plate was kept in a dark room for 30 minutes, and optical densities of the solutions were measured at 570 nm, with a microplate reader being used to release the coloured product into solution. Experiments were conducted in triplicate.

### 2.9. Cell Membrane Integrity Analysis (LDH Release)

Cells were seeded into a 96-well plate with a seeding density of 0.5 × 10^4^ cells per well (100 *μ*L/well) and incubated for 24 h. The medium was removed and replaced with various CaCO_3_ nanocrystals formulations. The plates were incubated at 37°C for 24, 48, and 72 h. After incubation, 2 *μ*L of lysis buffer was added into the positive control wells, and the plate was centrifuged (Thermo Fisher Scientific LPG) at 1,500 rpm for 10 min at 37°C. After centrifugation, 50 *μ*L of a membrane integrity assay reagent was added to the wells. The plates were again incubated for 10 min at 37°C while being protected from light. Subsequently, 30 *μ*L of 1 N HCl stop reagent was added into the wells, and the fluorescences of the samples were measured at 560 nm (excitation) and 590 nm (emission) on the microplate reader. The percentage of cytotoxicity was calculated with respect to the positive control wells where 100% lactate dehydrogenase (LDH) release was assumed.

### 2.10. Cell Uptake and Drug Release Investigations of DOX

MDA MB321 cells were seeded into a 6-well plate and allowed to grow for 3 days in 100% culture medium (DMEM). On reaching 80% cell confluence, the medium was replaced with 1 mL of fresh culture medium supplemented with 25 mM HEPES containing free DOX and CaCO_3_/DOX nanocrystals and incubated at 37°C for 6 h. The cells were washed three times with PBS before analysis by confocal microscopy.

Fluorescence images were made using the autofluorescence of DOX excited at 488 nm. The fluorescence emission (between 565 and 630 nm) was visualised by confocal microscopy (Olympus Confocal Microscope System).

### 2.11. Morphological Examination

Fluorescence microscopic observation is one of the most reliable tests for determining and quantifying cell viability and cell death through the use of different cell stains.

### 2.12. Acridine Orange (AO) and AO and Propidium Iodide Double Staining

The cells were seeded in 6-well plate for 1 day, followed by treatment with different concentrations of free DOX and CaCO_3_/DOX nanocrystals for a period of 24 h. The medium was removed and washed 2 times with PBS. The cells were stained with Acridine orange dye (1 *μ*g/mL) for 5 minutes and examined with an inverted fluorescence microscope. A similar procedure was carried out for double staining with an Acridine orange dye (1 *μ*g/mL)/propidium iodide (0.5 *μ*g/mL) mixture for 10 minutes.

## 3. Results and Discussion

**Figure 1:**
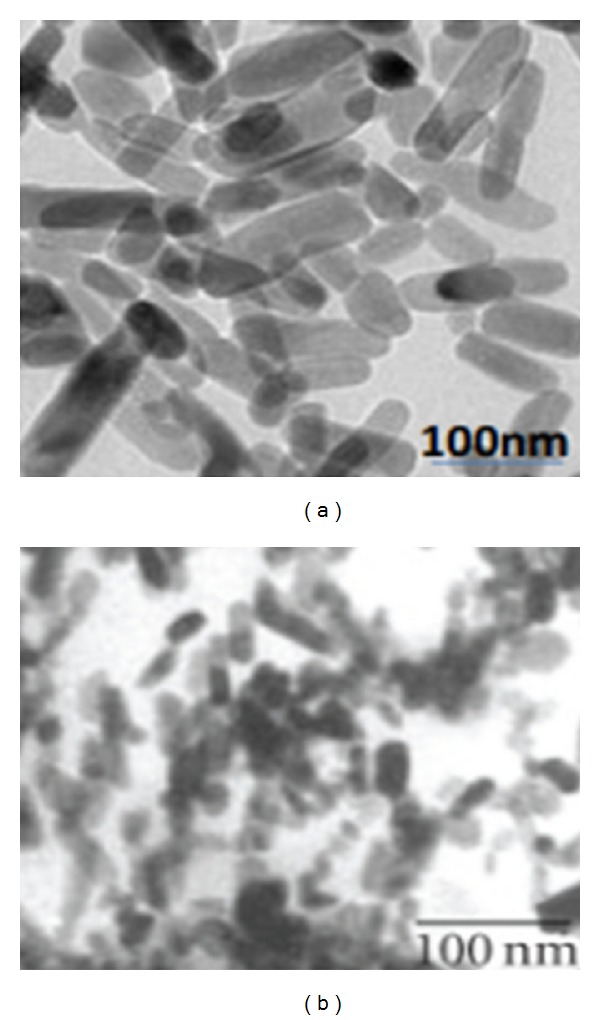
TEM micrographs of rod-shaped CaCO_3_ nanocrystals (a) before drug loading and (b) after drug loading.

Images obtained by transmission electron microscopy (TEM) indicated that the calcium carbonate nanocrystals demonstrate a perfect rod-shaped morphology with a uniformly distributed average size of 35 nm to 60 nm. Particles before loading of drugs are shown in Figure 1(a), while Figure 1(b) indicates particles after loading, and the rod shapes are maintained. The observed rod shapes of the particles are the naturally occurring morphology of the calcium carbonate aragonite polymorph, which is the specific target of our research. 

**Figure 2:**
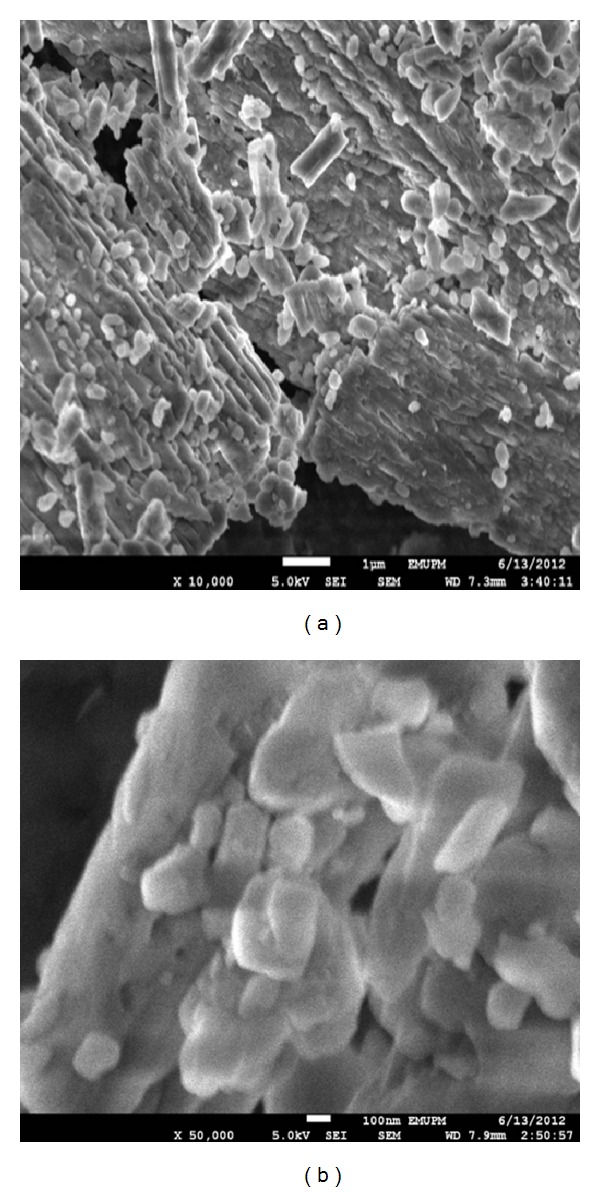
FESEM micrographs of rod-shaped CaCO_3_ nanocrystals (a) before drug loading and (b) after drug loading.

Surface characterisation of the CaCO_3_ nanocrystals by field emission scanning electron microscopy (FESEM) also displayed the rod shape of the particles before (Figure 2(a)) and after the drug loading (Figure 2(b)), confirming the structure shown by TEM (Figures 1(a) and 1(b)). Importantly, particle shapes were maintained throughout the experiment. Recent reports indicated that rod-shaped nanocarriers are more effective and selective in the delivery of agents to cancer cells than spherically shaped carriers [14].

Particle charge, or zeta potential, and size distribution were analysed by zetasizer (Nano ZS, in Malvern Instruments). The measurements employed dynamic light scattering by utilising the comparative faster thermal motion of small particles in comparison to larger particles to determine particle size. Dynamic light scattering indicated that particles were approximately within 100 nm in size, similar to the individual particles size determined by TEM (Figure 1(a)), while the particle zeta potentials were found to be negative.

### 3.1. Drug Loading and Encapsulation Efficiency

Drug loading and encapsulation are essential parameters in any efficient drug delivery system. Due to the porous nature of the synthesised calcium carbonate nanocrystals, as seen in the FESEM micrograph (Figure 2(b)), drugs may be loaded, or encapsulated, into the inner pores of the particles. As observed from the data, drug loading has a significant effect on the drug encapsulation efficiency; higher drug loading reduced the encapsulation efficiency. The greater the amount of drug loaded into the CaCO_3_ nanocrystals is, the greater the percentage of drug will be lost to the surrounding medium. This phenomenon has been investigated by other researchers [15, 16]. The results of drug loading and encapsulation efficiency are summarised in Table 1.

### 3.2. Elemental Analyses of CaCO_3_/DOX Nanocrystals

**Figure 3:**
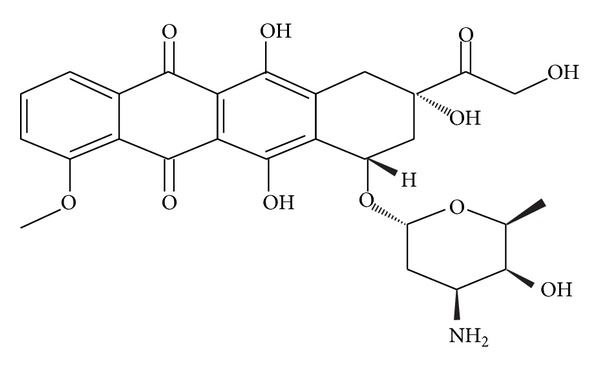
Chemical structure of doxorubicin (C_27_H_29_NO_11_).

Elemental analysis is another method of quantitative and qualitative determination for the presence of DOX in calcium carbonate nanocrystals. The results of elemental compositions of CaCO_3_/Dox nanocrystal were summarised and presented in Table 2. The presented data signifies the presence of nitrogen from amine group (NH_2_) and hydrogen from the hydroxyl (OH) group of doxorubicin (C_27_H_29_NO_11_) as shown in the structure (Figure 3). Presence of N and H in the drug loaded of CaCO_3_ nanocrystal comfirm the interclation of drug into the nanocrystal, since pure or unloaded calcium carbonate does not contained such elements. therefore, elemetal composition of CaCO_3_ are Ca, O and C only, any addition of N and H may definately be from the drug used, thus confirming the intercalation or encapsulation of DOX into the calcium carbonate nanocrystal. 

### 3.3. Release Studies of Doxorubicin

**Figure 4:**
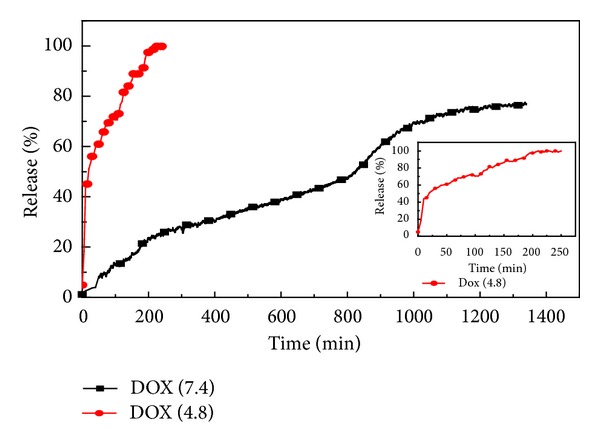
Doxorubicin release profiles in PBS, pH 4.8 and 7.4.

The* in vitro* evaluation of doxorubicin from the loaded CaCO_3_ nanocrystals was conducted as stated in the methodology. Therapeutic effectiveness is closely related to the release of drugs from carrier systems. *In vitro* release of the drug was studied by simulating the different pH regimes of tumours (acidic environment, pH 4.8) and blood/normal tissue (pH 7.4). One of the most promising characteristics of our loaded calcium carbonate nanocrystals is the ability to release DOX in response to changes in pH. The graph in Figure 4 shows the release of DOX at normal physiological pH (7.4), where approximately 80% of the loaded DOX was released within 1200 minutes, whilst when exposed to a pH of 4.8, 80% of the incorporated DOX was released in 50 minutes; the observed release evidently indicated that the drug may be delivered without affecting normal cells. Also an initial burst-release of up to 40% of the DOX was recorded within 10 to 15 min at pH 4.8, followed by sustained release of encapsulated DOX culminating in complete unloading by 250 min. The sustained release of 100% of DOX in 250 min may induce apoptosis and prevent cancer metastasis at the early stage. 

**Figure 5:**
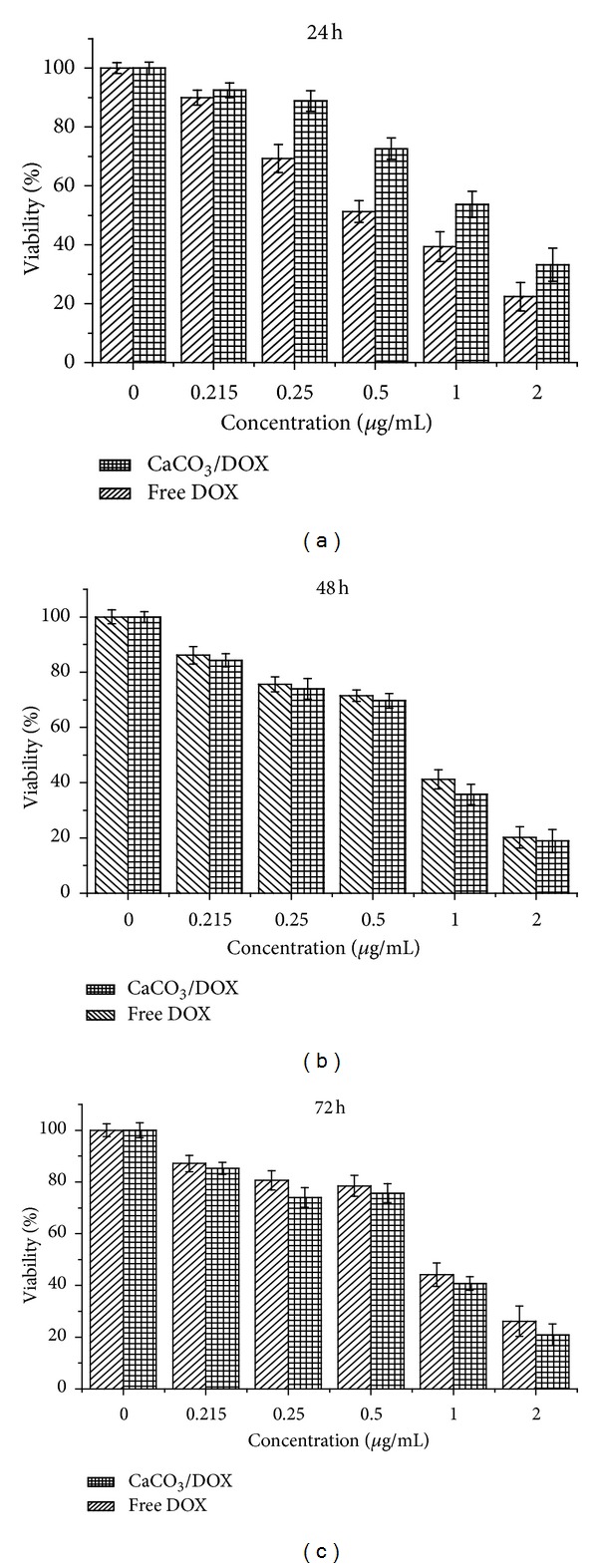
*In vitro* cytotoxicity study of MDA MB231 cells after 24, 48, and 72 hours of exposure to free DOX and the CaCO_3_/DOX nanocrystals.

Figure 4 presents the effects of free DOX and CaCO_3_/DOX nanocrystals against the MDA MB 231 breast cancer cell line. The results demonstrated both concentration and time-dependent toxicity; increases in both concentration and time increased the inhibitory effect on cells growth. Cells were treated for 72 h with free DOX and CaCO_3_/DOX. Monitored cell inhibition within 24 h indicated the higher toxicity of free DOX compared to CaCO_3_/DOX (Figure 5), while at 42 h no significant difference was observed in the inhibition rate by the two delivery methods (*P* > 0.05). At 72 h the CaCO_3_/DOX nanocrystals showed greater toxicity to cells than free DOX (*P* < 0.5). The explanation of this trend may lay with the fact that free DOX has immediate direct contact with the MDA MB 231 cells, which may initially induce a substantial inhibition of cell growth that diminishes with increasing time. Also, tumour uptake of free DOX takes place through passive diffusion, which may result in trapping the drug at the P-gap junction and adverse effects in normal cells. In the case of CaCO_3_/DOX, the drug is released in a time-dependent manner from the CaCO_3_ nanocrystals before exerting its effects on the cells. Therefore, as the rate of drug release increases, the concentration of DOX also increases, giving the observed time-dependent nature of cell-growth inhibition. The mechanism of CaCO_3_/DOX delivery to tumour cells is nonspecific endocytosis, thereby reducing cytosolic effect of free DOX for the P-gp pumping action. This mechanism may evade the effect of multidrug resistance proteins, present in cancer cells [17, 18]. 

A similar trend was observed in the modified neutral/trypan blue exclusion assay after 24 h of treatment. Free DOX is more toxic to MDA MB 231 cells, as indicated by MTT, for the first 24, but at 42 and 72 of treatment the CaCO_3_/DOX nanocrystals showed higher toxicity than free DOX, corresponding to 72 h of  MTT treatment.

### 3.4. LDH Release

The presence of elevated lactate dehydrogenase (LDH) activity in the cell supernatant is a popular method for the assessment of the *in vitro* cytotoxicity potential of a compound, extract, or formulation. Membrane integrity was analysed by measuring the amount of LDH released, which is proportional to the number of cells damaged or lysed. 

**Figure 6:**
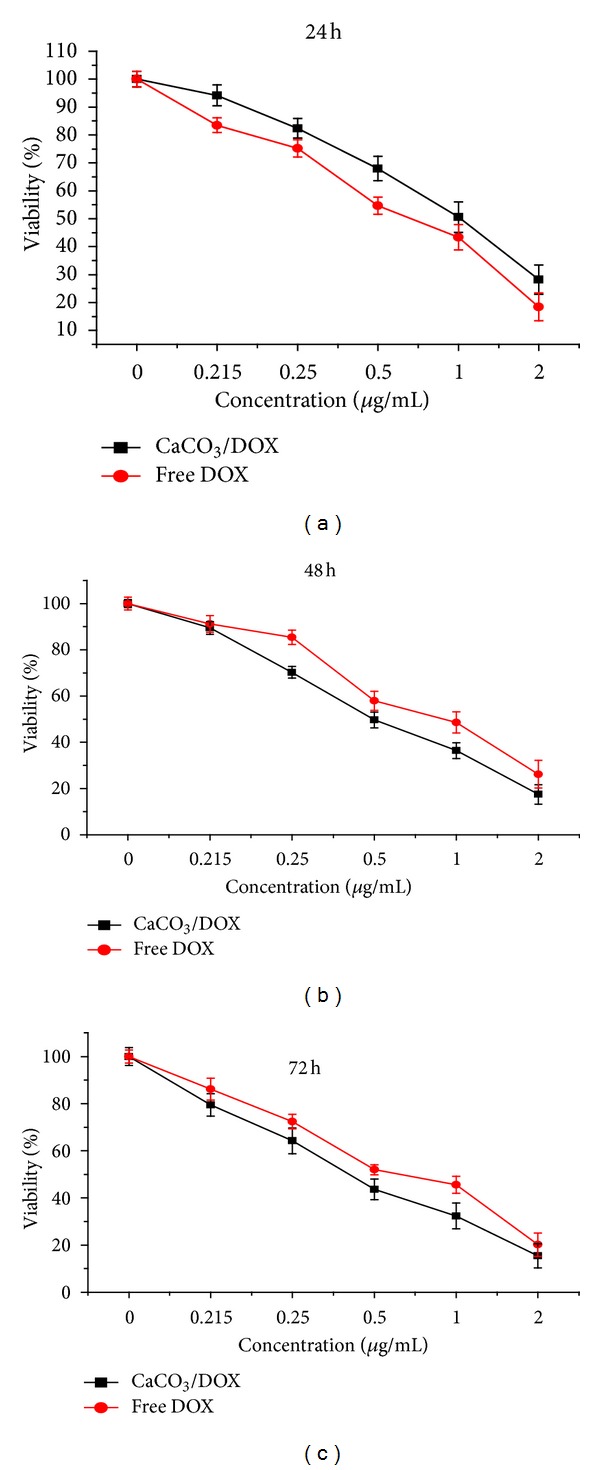
Modified neutral red/trypan blue exclusion assay. MDA MB231 cells after 24, 48, and 72 h of exposure to free DOX and CaCO_3_/DOX nanocrystals.

**Figure 7:**
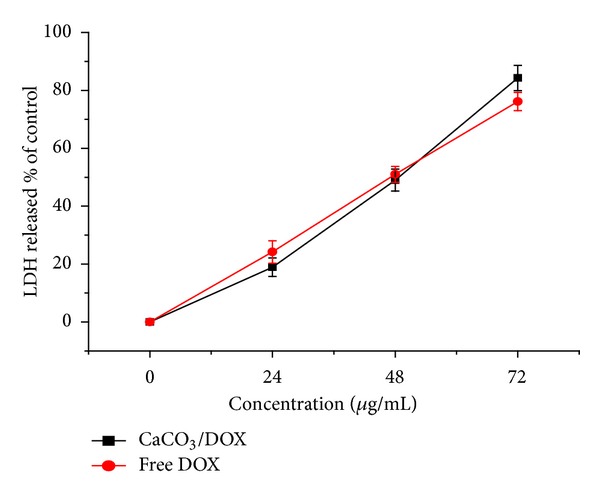
LDH release of MDA MB231 cells after 24, 48, and 72 hours of exposure to free DOX and the CaCO_3_/DOX nanocrystals.

The LDH released in MDA MB231 cells exposed to free DOX was higher after 24 and 48 h of treatment compared to CaCO_3_/DOX nanocrystals within the same period, likely as a result of slow release. Surprisingly, higher LDH levels were observed for CaCO_3_/DOX nanocrystals after 72 h (Figure 6). Apparently, the total release of  DOX was significantly higher in the loaded CaCO_3_ (CaCO_3_/DOX nanocrystals) than that of free DOX. This result agrees with our MTT neutral red/trypan blue exclusion assay (Figures 5 and 6), while the same mechanism may be used to explain the increase in LDH released at 72 h by the CaCO_3_/DOX nanocrystals Figure 7.

**Figure 8:**
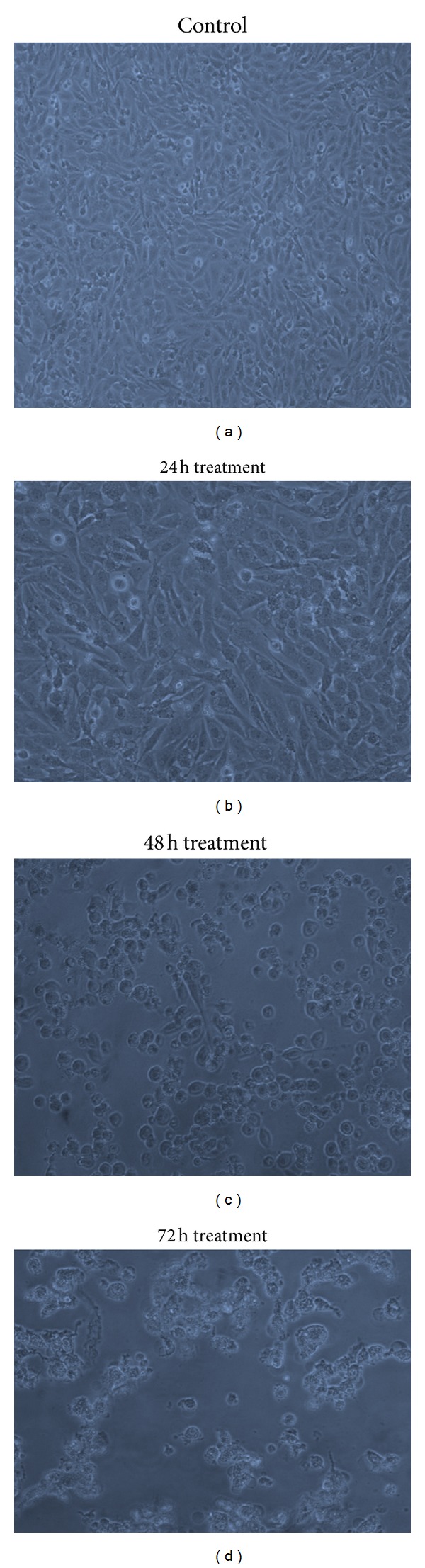
Images of MDA MB231 cells treated with different agents at 24, 48, and 72 h of incubation.

The morphological observation of MDA MB 231 cells treated with IC_50_ concentration of CaCO_3_/DOX nanocrystals for periods of 24, 48, and 72 h revealed that at 24 h some cells became rounded and detached from the surface of the flask. At 48 and 72 h the majority of the treated cells were rounded and many were suspended in the culture media, while others showed visible blabbing characteristics. The numbers of cells were reduced compared to those of the control. This study showed effective delivery of anticancer drug (DOX) and inhibition of cell growth by the drug-loaded CaCO_3_ nanocrystals (Figure 8).

### 3.5. Cellular Uptake of CaCO_3_/DOX and Free DOX

**Figure 9:**
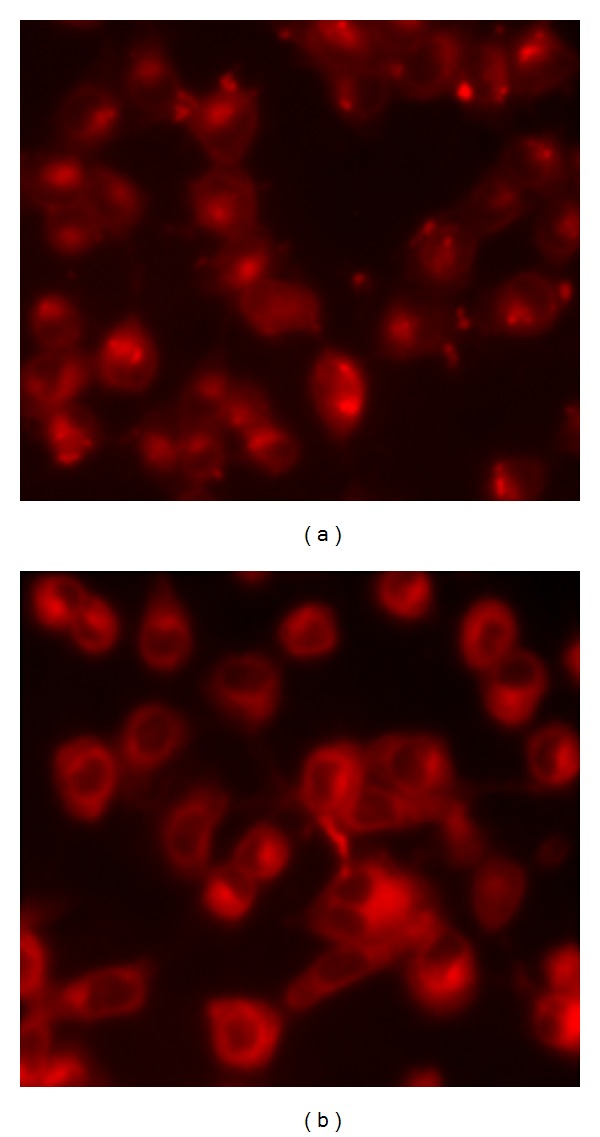
After 8  of treatment with free DOX (a) and CaCO_3_ nanocrystal/DOX (b).

The mechanisms of intracellular uptake of free DOX and CaCO_3_/DOX nanocrystals are quite different. As shown in Figures 9(a) and 9(b), it was observed that DOX was transported into the cellular compartment; the mode of transportation differs.  Figure 9(a) showed the direct delivery of free DOX into the cells cytoplasm by a very strong red fluorescence of free DOX in cellular nuclei. This indicated a rapid intercalation of  DOX into the chromosomal DNA by passive diffusion [16].  Figure 9(b) shows delivery of  DOX by CaCO_3_/DOX nanocrystals, where the florescence nature of Dox was detected in abundance in the cytoplasm with a very little found in the nuclei. This verifies the differences in the delivery mechanisms of free DOX and CaCO_3_/DOX nanocrystals.  Figure 9(b) demonstrates the trapping of  DOX in the cellular endosomal compartment, which consists of a complex set of vesicles and tubules extending from the cell periphery (early endosomes) to the perinuclear region. This is the region where the ligand is dissociated from the receptor, and decisions are made about whether to send receptor for degradation or to recycle it to the cell surface [18, 19]. 

**Figure 10:**
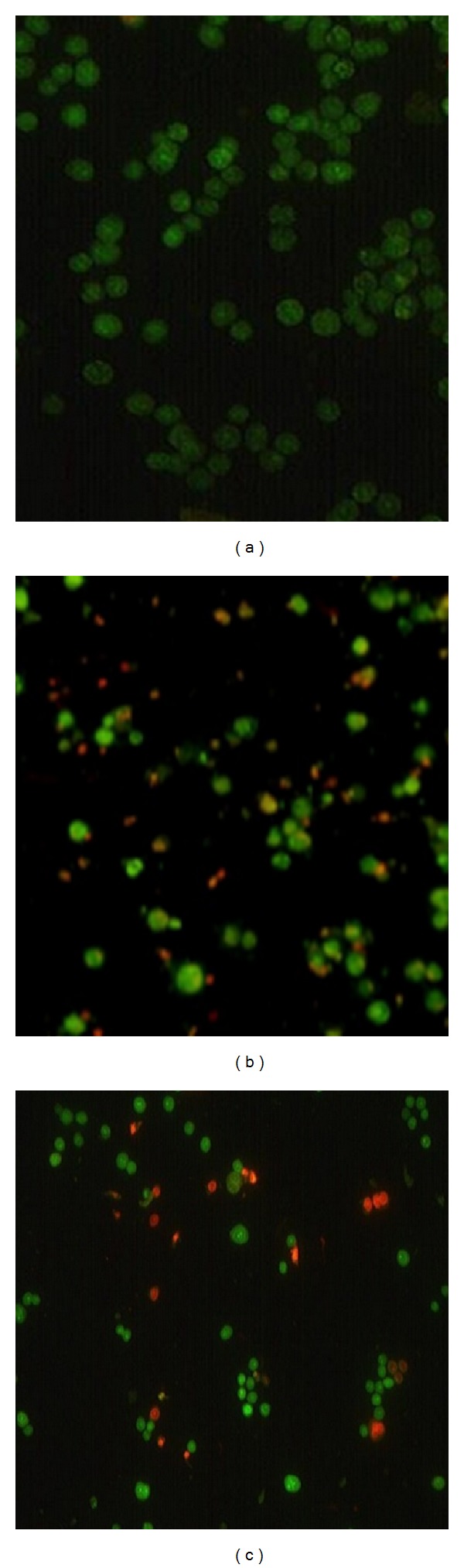
Fluorescent images of MDA MB231 cells stained with Acridine orange (AO): (a) control, (b) treated with free DOX, and (c) CaCO_3_/DOX nanocrystals.

Morphological analysis of dead and live cells by Acridine orange (AO) and propidium iodide (PI) double staining is an excellent method for the accurate measurement of cell viability as shown in Figure 10. AO is a lipid soluble dye, permeable to both live and dead cells, that stains all nucleated cells green, while propidium iodide (PI) enters only dead cells, with compromised membranes, staining them red. AO/PI staining is used to differentiate between the necrotic and apoptotic modes of cells death. Viable cells have round nuclei and are stained green, while cells undergoing apoptotic death are stained green with fragmented DNA and cell blabbing. The fragmented DNA of necrotic and late apoptotic cells are stained red and orange.

## 4. Conclusions

We developed and demonstrated a bio-based calcium carbonate nanocrystal carrier that can effectively deliver a wide range of therapeutic drugs with pH-sensitive properties. The carrier has the capacity for large loads of anticancer drugs and is able to deliver these agents selectively to cancer cells with high specificity, achieving effective cancer cell death without inducing nonspecific toxicity. Slow release was observed at normal physiological pH (7.4) with a faster release at acidic pH (4.8) simulating tumour microenvironment. This study indicated that the DOX-loaded CaCO_3_ nanocrystals are promising materials in the delivery of anticancer drugs.

## Figures and Tables

**Table 1:** Drug loading concentrations, loading content and encapsulation efficiency.

Samples	Weight of nanocrystal (mg)	Weight of drug (mg)	Loading content (%)	Encapsulation efficiency (%)
CaCO_3_ (1)	100	2	4.8	96
CaCO_3_ (2)	100	4	9.4	82
CaCO_3_ (3)	100	6	13.7	64

**Table 2:** Elemental compositions of drug loaded calcium carbonate nanocrystals.

Samples	% C	% H	% N	N/H
CaCO_3_/Dox	13.128	0.8954	0.6447	1.39
Doxorubicin	53.91	5.21	2.22	2.34

## References

[1] Thurston Devid E (2007). *Chemistry and Pharmacology of Anticancer Drugs*.

[2] Mundy GR (2002). Metastasis to bone: causes, consequences and therapeutic opportunities. *Nature Reviews Cancer*.

[3] Iddon J, Byrne G, Bundred NJ (1999). Bone metastasis in breast cancer: the role of parathyroid hormone related protein. *Surgical Oncology*.

[4] Hortobagyi GN (2002). Novel approaches to the management of bone metastases in patients with breast cancer. *Seminars in Oncology*.

[5] Brown JE, Coleman RE (2002). The present and future role of bisphosphonates in the management of patients with breast cancer. *Breast Cancer Research*.

[6] Bäuerle T, Merz M, Komljenovic D, Zwick S, Semmler W (2010). Drug-induced vessel remodeling in bone metastases as assessed by dynamic contrast enhanced magnetic resonance imaging and vessel size imaging: a longitudinal in vivo study. *Clinical Cancer Research*.

[7] Thobe MN, Clark RJ, Bainer RO, Prasad SM, Rinker-Schaeffer CW (2011). From prostate to bone: key players in prostate cancer bone metastasis. *Cancers*.

[8] El Hazzat J, El-sayed MEH (2010). Advances in targeted breast cancer therapy. *Current Breast Cancer Reports*.

[9] Cho K, Wang X, Nie S, Chen Z, Shin DM (2008). Therapeutic nanoparticles for drug delivery in cancer. *Clinical Cancer Research*.

[10] Sharif H, Yamamoto H, Chowdhury EH (2013). Fabrication and intracellular delivery of doxorubicin/ carbonate apatite nanocomposites: effect on growth retardation of established colon tumor. *PLoS ONE*.

[11] Rameshwar P, Portilla-Arias J, Ding H (2012). Cellular delivery of doxorubicin via pH-controlled hydrazone linkage using multifunctional nano vehicle based on poly(*β*L-malic acid). *International Journal of Molecular Sciences*.

[12] Awang-Hazmi AJ, Zuki ABZ, Nordin MM, Jalila A, Norimah Y (2007). Mineral composition of the cockle (*Anadara granosa*) shells of west coast of peninsular Malaysia and its potential as biomaterial for use in bone repair. *Journal of Animal and Veterinary Advances*.

[13] Kamba AS, Ismail M, Ibrahim TAT, Zakaria ZAB (2013). Synthesis and characterisation of calcium carbonate aragonite nanocrystals from cockle shell powder (*Anadara granosa*). *Journal of Nanomaterials*.

[14] Barua S, Yoo J-W, Kolhar P, Wakankar A, Gokarn YR, Mitragotri S (2013). Particle shape enhances specificity of antibody-displaying nanoparticles. *Proceeding of the National Academy of Sciences of the United State of America*.

[15] Wang S-B, Chen A-Z, Weng L-J, Chen M-Y, Xie X-L (2004). Effect of drug-loading methods on drug load, encapsulation efficiency and release properties of alginate/poly-L-arginine/chitosan ternary complex microcapsules. *Macromolecular Bioscience*.

[16] Zhao Q, Han B, Wang Z, Gao C, Peng C, Shen J (2007). Hollow chitosan-alginate multilayer microcapsules as drug delivery vehicle: doxorubicin loading and in vitro and in vivo studies. *Nanomedicine*.

[17] Dong Z, Liu C-J, Zhuo R-X, Cheng S-X (2012). Alginate/CaCO_3_ hybrid nanoparticles for effcient co delivery of antitumor gene and drug. *Molecular Pharmaceutics*.

[18] Upadhyay KK, Bhatt AN, Mishra AK (2010). The intracellular drug delivery and anti tumor activity of doxorubicin loaded poly(*γ*-benzyl l-glutamate)-b-hyaluronan polymersomes. *Biomaterials*.

[19] Detlev G, Klaus R, Detlev G, Klaus R (2013). Endosomal compartment. *Encyclopedic Reference of Genomics and Proteomics in Molecular Medicine*.

